# Adaptive laboratory evolution and shuffling of *Escherichia coli* to enhance its tolerance and production of astaxanthin

**DOI:** 10.1186/s13068-022-02118-w

**Published:** 2022-02-16

**Authors:** Qian Lu, Xiao-Ling Zhou, Jian-Zhong Liu

**Affiliations:** Institute of Synthetic Biology, School of Life Sciences, Sun Yat-Sen University, Guangzhou, 510275 People’s Republic of China

**Keywords:** Astaxanthin, Genomic engineering, *Escherichia coli*, Tolerance, Biosynthesis

## Abstract

**Background:**

Astaxanthin is one of the strongest antioxidants in nature and has been widely used in aquaculture, food, cosmetic and pharmaceutical industries. Numerous stresses caused in the process of a large scale-culture, such as high acetate concentration, high osmolarity, high level of reactive oxygen species, high glucose concentration and acid environment, etc., limit cell growth to reach the real high cell density, thereby affecting astaxanthin production.

**Results:**

We developed an adaptive laboratory evolution (ALE) strategy to enhance the production of chemicals by improving strain tolerance against industrial fermentation conditions. This ALE strategy resulted in 18.5% and 53.7% increases in cell growth and astaxanthin production in fed-batch fermentation, respectively. Whole-genome resequencing showed that 65 mutations with amino acid substitution were identified in 61 genes of the shuffled strain *Escherichia coli* AST-4AS. CRISPR interference (CRISPRi) and activation (CRISPRa) revealed that the shuffled strain with higher astaxanthin production may be associated with the mutations of some stress response protein genes, some fatty acid biosynthetic genes and *rppH*. Repression of *yadC, ygfI* and *rcsC*, activation of *rnb, envZ* and *recC* further improved the production of astaxanthin in the shuffled strain *E. coli* AST-4AS. Simultaneous deletion of *yadC* and overexpression of *rnb* increased the production of astaxanthin by 32% in the shuffled strain *E. coli* AST-4AS.

**Conclusion:**

This ALE strategy will be powerful in engineering microorganisms for the high-level production of chemicals.

**Supplementary Information:**

The online version contains supplementary material available at 10.1186/s13068-022-02118-w.

## Background

Astaxanthin (3,3′-dihydroxy-β-carotene-4,4′-dione), a red-color carotenoid, is one of the strongest antioxidants in nature [1], exhibiting tremendous potential for applications in healthcare and pharmaceuticals due to its antioxidant [2], anti-inflammatory [3] and anti-cancer activity [4]. It has been widely used in aquaculture, food, cosmetic and pharmaceutical industries [5]. According to the analysis of Stratistics Market Research Consulting (MRC), the global astaxanthin market is accounted for $695.94 million in 2020 and is expected to reach $1356.47 million by 2028 growing at a compound annual growth rate of 8.7% [6]. Currently, commercial astaxanthin production relies on two routes, chemical synthesis and extraction from nature producers (such as *Haematococcus pluvialis* algae) [6]. However, the former route of production is not sustainable and poses concerns in health and food safety, while the latter is very costly (> $7000 per kg) [7].

To lower the cost, many studies focused on the production of astaxanthin from an inexpensive carbon source using microbes. *Escherichia coli* [8–17], *Saccharomyces cerevisiae* [18–24], *Corynebacterium glutamicum* [25] and *Yarrowia lipolytica* [26] have been used as host strains for astaxanthin production by the introduction of the astaxanthin biosynthesis pathway. In our study [9], we engineered an *E. coli* that produced 7.4 mg/g dry cell weight (DCW) of astaxanthin as the predominant carotenoid (96.6%) by coordinately expression of β-carotene hydroxylase (CrtZ) and ketolase (CrtW). Further morphology and oxidative stress engineering increased the yields to 11.92 mg/g DCW via [17]. The highest astaxanthin yield in *E. coli* and *S. cerevisiae* was 15.1 mg/g DCW [15] and 13.8 mg/g DCW [18] so far, respectively. In addition, it has ample space to further improve if compared to lycopene, carotene and zeaxanthin (> 23 mg/gDCW) [27–29].

It has been demonstrated that carotenoids are accumulated in the cellular membrane of *E. coli* [30], and high cell density culture is favorable for the production of astaxanthin. However, high cell density culture can impose numerous stresses on cells, including high acetate concentration, high osmolarity (NaCl concentration) (due to the need to neutralize acids produced as fermentation products or by-products), high level of reactive oxygen species, high glucose concentration and acid environment, etc. These factors limit the cell growth to reach the real high cell density in industrial fermentations. Thus, increasing the tolerance of *E. coli* to industrial cultural conditions may be a strategy for improving the production of astaxanthin.

Adaptive laboratory evolution (ALE) and genome shuffling are widely used and highly effective tools in tolerance engineering for creating industrial strains resistant to some industrially relevant stress [31–35]. ALE can be used to rapidly evolve and screen robust mutants, and omics analysis can be subsequently be used to investigate the relationship between the strain genotype and phenotype, thereby guiding inverse metabolic engineering. Error-prone whole-genome shuffling (ep-WGS) is a simple, rapid, efficient shuffling technology, which does not use mutagens and protoplast fusion [36]. It has been successfully applied to improve acid tolerance of *Lactobacillus pentosus* [36], butanol of tolerance of *E. coli* [37], shikimate production [35].

To explore the relationship between the genotype and phenotype, a CRISPR interference (CRISPRi) and activation (CRISPRa) approach based on CRISPR-Cas-SoxS system was developed [38]. In this system, genes can be simultaneously activated and repressed. Moreover, we can rapidly investigate the function of many genes because constructing the scRNA expressing vector for simultaneous activation of repression of all genes is rather time-consuming.

In this study, we first applied ALE after atmospheric and room temperature plasma (ARTP) mutagenesis and ep-WGS to enhance the tolerance of *E. coli* against industrial cultural conditions and the production of astaxanthin. Then, whole-genome resequencing was carried out to reveal the relationship between the strain genotype and phenotype.

## Results and discussion

### ALE

**Fig. 1:**
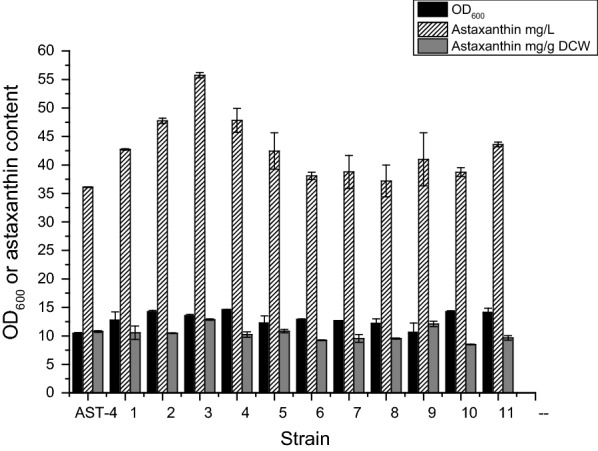
Production of astaxanthin by the selected adaptive evolved strains after ARTP mutation. The data represent the means of three replicates, and error bars indicate standard deviations

High cell density culture is favorable for the production of astaxanthin. However, high cell density culture can cause high acetate concentration, high osmolarity (NaCl concentration), high level of reactive oxygen species, the high acid environment. These factors limit the cell growth to reach the real high cell density. Thus, *E. coli* AST-4 was first mutated by ARTP, and then mutants were subsequently cultured in LB medium with gradually increased concentrations of NaAc, NaCl and H_2_O_2_, and decreased pH value, until reached 5 g/L, 0.3 M, 2 mM and 5.5, respectively. Inspired by astaxanthin can efficiently diffuse through the cell membrane, Zhang et al. developed a colorimetry of culture media (measured the absorbance at 515 nm) for the screening of high astaxanthin-producing strains [15]. We also demonstrated that the intracellular production of astaxanthin was correlated with absorbance at 515 nm of the growth media (Additional file 1: Fig. S1). Thus, the colorimetrical method was used to screen the library. A total of 380 colonies with the reddest color on plates were selected for the deep-well microplate culture (Additional file 1: Fig. S2). Eleven strains with a higher OD_515_ value than the starting strain *E. coli* AST-4 were selected for further shake flask fermentation (Fig. 1). All 11 mutants grew better than the starting strain (AST-4). Of them, the mutant No.3 exhibiting the highest astaxanthin productivity, was selected for further analysis and renamed AST-4A. The astaxanthin content, concentration and biomass of this strain were increased by 19%, 35% and 19% compared with the starting strain *E. coli* AST-4, respectively.

**Fig. 2:**
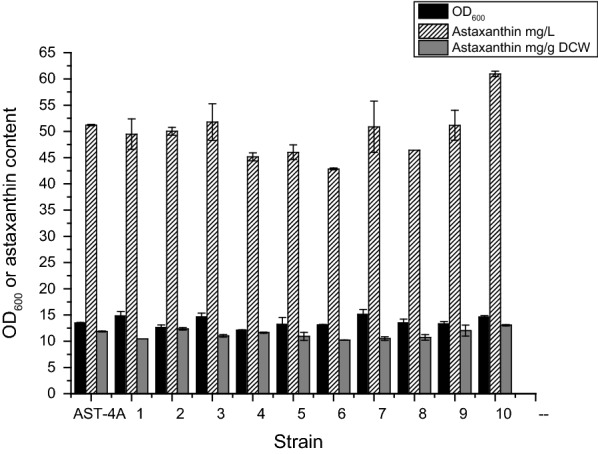
Production of astaxanthin by the selected shuffled strains. The data represent the means of three replicates, and error bars indicate standard deviations

Subsequently we applied ep-WGS for *E. coli* AST-4A to further improve the production of astaxanthin. A total of 400 colonies with the reddest color on plates were selected for the deep-well microplate culture (Additional file 1: Fig. S3). Ten strains with a higher OD_515_ value than *E. coli* AST-4A were selected for further shake flask fermentation (Fig. 2). Strain No. 10 produced a higher level of astaxanthin and grew well than the starting strain *E. coli* AST-4A, and was renamed as *E. coli* AST-4AS.

**Fig. 3:**
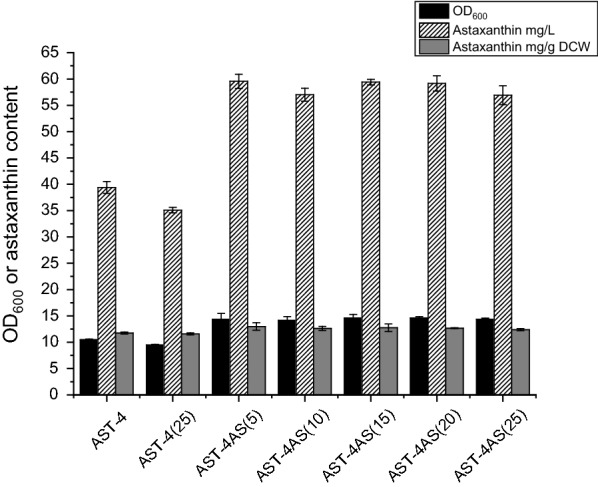
Genetic stabilities of the shuffled strain. The number in parentheses in the strain name denotes the number of generations. The data represent the means of three replicates, and error bars indicate standard deviations

Then we assess the genetic stability of the shuffled strain. *E. coli* AST-4AS was cultured by continuous subculture. As shown in Fig. 3, the astaxanthin production of the genome shuffled strain was not significantly different after 25 generations, indicating that the genome shuffled strain AST-4AS shows good genetic stability. However, the yield of astaxanthin of the parent strain *E. coli* AST-4 decreased by about 10% after 25 generations (Fig. 3). It indicates that the shuffled strain AST-4AS had better genetic stability than the parent strain *E. coli* AST-4. We can also find that the genome shuffled strain produced a 51.2% higher level of astaxanthin than the parent strain *E. coli* AST-4.

**Fig. 4:**
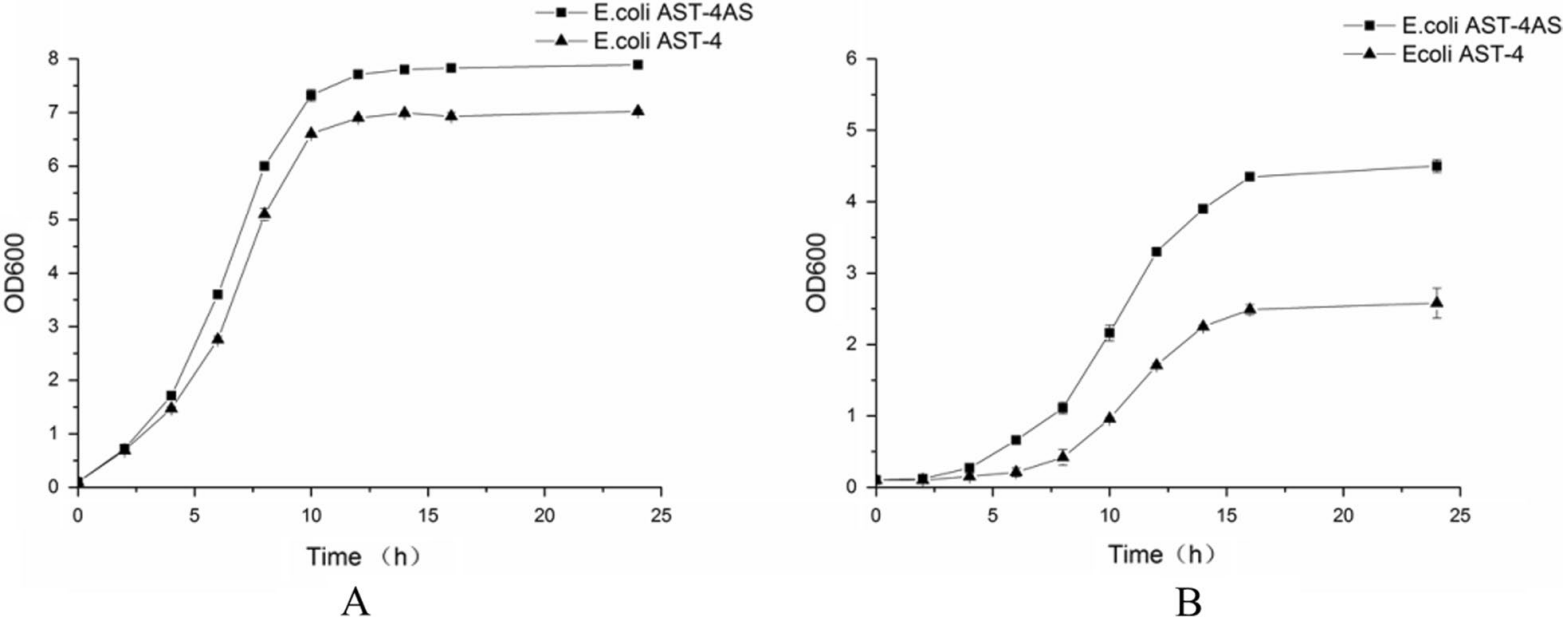
Growth of *E. coli* AST-4AS and *E. coli* AST-4 in the LB medium (**A**) and the LB medium with the selection stress conditions (5 g/L NaAc, 0.3 M NaCl and 2 mM H_2_O_2_, pH 5.5) (**B**). The data represent the means of three replicates, and error bars indicate standard deviations

To evaluate the tolerance of the shuffled strain, the growth of the genome shuffled strain was compared with the parent strain *E. coli* AST-4. As shown in Fig. 4, the shuffled strain *E. coli* AST-4AS grew better than the parent strain *E. coli* AST-4 in the absence of the selected stress conditions for high cell density culture. The biomass (OD_600_ value) of *E. coli* AST-4AS was 12.4% higher than that obtained by the parent strain *E. coli* AST-4 in the absence of the selected stress conditions. In the presence of the selected stress conditions, the shuffled strain *E. coli* AST-4AS grew well. The highest OD_600_ value of the shuffled strain *E. coli* AST-4AS was 4.50, which is 74.4% higher than that of the parent strain *E. coli* AST-4.

**Fig. 5:**
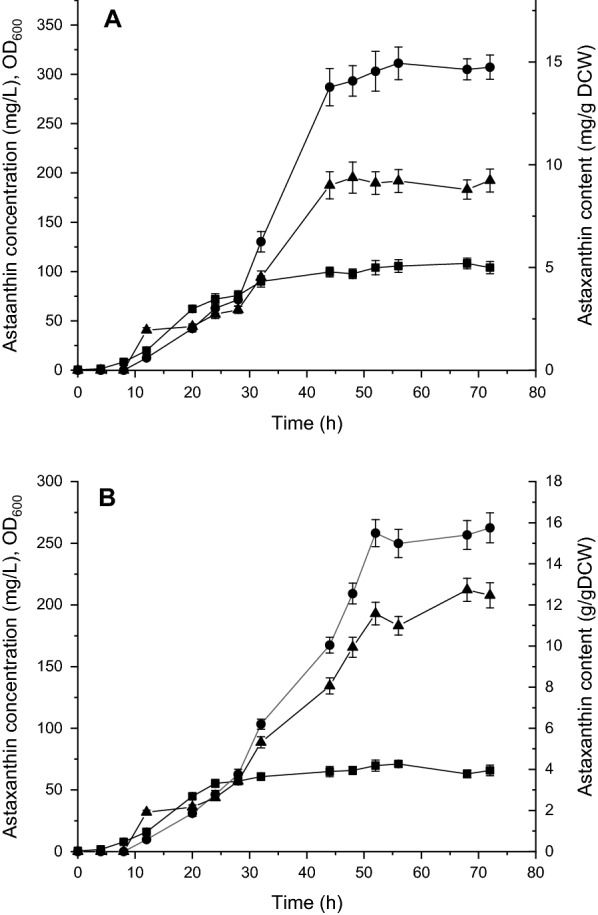
Fed-batch fermentation of *E. coli* AST-4AS (**A**) and *E. coli* AST-4 (**B**) in a 2-L bioreactor. (Filled square) OD_600_; (Filled circle) Astaxanthin concentration; (filled triangle) Astaxanthin content. The data represent the means of three replicates, and error bars indicate standard deviations

Fed-batch fermentation was applied to investigate the production in the mutant strain in a 2-L bioreactor. As shown in Fig. 5, the highest OD_600_ value of the genome shuffled strain *E. coli* AST-4AS were 100.37 ± 0.25, which was 18.5% higher than that of *E. coli* AST-4. *E. coli* AST-4AS produced astaxanthin of 311.20 ± 0.36 mg/L and 9.21 ± 0.12 mg/g DCW with a productivity of 5.55 mg/L/h, which was a 53.7% increase in the astaxanthin titer compared to *E. coli* AST-4. The astaxanthin titer obtained by *E. coli* AST-4AS is lower than the highest value (1.18 g/L) ever reported [16]. The highest astaxanthin titer was obtained by high cell-density culture with OD600 of 469 [16]. However, the astaxanthin content (9.21 ± 0.12 mg/g DCW) obtained by *E. coli* AST-4AS is higher than that (7.8 mg/g DCW) reported in this literature [16]. Moreover, the astaxanthin ratio in the total carotenoid reached about 100% in *E. coli* AST-4AS (Additional file 1: Fig. S4), which is much higher than that (53%) reported in this literature [16]. These indicate that the fed-batch fermentation conditions should be optimized for increasing the cell density, thereby enhancing the astaxanthin titer.

### Whole-genome resequencing

To investigate the relationship between the genotype and the phenotype of the genome shuffled strain *E. coli* AST-4AS, the whole genome of *E. coli E. coli* AST-4AS was re-sequenced. A total of 91 single nucleotide variants (SNVs) and 33 insertion/deletions (InDels) were identified in *E. coli* AST-4AS (Table 1 and Additional file 1: Table S1). Of them, 65 mutations located in 61 genes cause the substitutes of amino acids.

**Table 1:** SNV and InDel mutants in *E. coli* AST-4AS

Type	Amounts of mutant	Amounts of gene
SNV	91	
Synonymous SNV	27	21
Nonsynonymous SNV	51	48
Stop gaining SNV	3	3
Start lost SNV	1	1
Intergenic region	9	
InDel	33	
Frameshift variant	10	9
Intergenic region	23	

Mapped reads ratio of 98.83%, covered bases of 4,641,652 bp, coverage of 99.98%, unique read ratio of 97.11, the SNVs and InDels calling were performed with the stand_call_conf 50 and stand_emit_conf 10.0

To reveal the effects of each mutation on astaxanthin production, we first applied the CRISPR-Cas-SoxS system [38] for repressing the mutated genes in *E. coli* AST-4. pBbB2K-dCas9*-MCPSoxS and pTargetA-X were co-transferred into *E. coli* AST-4. Astaxanthin production was analyzed and compared to the control strain harboring the empty vector pTargetA. The results are presented in Table 2 and Additional file 1: Fig. S5. The repression of 29 genes increased the production of astaxanthin by 5–63%. The repressions of some genes related to stress response, such as *yadC, lon, cusB, acnA, rcsC* and *ygfI*, increased the production of astaxanthin. It was also found that the repressions of some genes involved in fatty acid biosynthesis, such as *fabI* and *fabR*, improved the production of astaxanthin. The repressions of them can provide more acetyl-CoA, ATP and NADPH for the production of astaxanthin. The repression of *rppH* increased the production of astaxanthin by 21%. The *rppH* encodes RNA pyrophosphohydrolase. It has been reported that the RppH pyrophosphohydrolase activity deficiency increased the mRNA levels of glycolytic genes, thereby allowing fast growth [39]. Thus, we guess that the repression of *rppH* caused the increase in the mRNA levels of glycolytic genes, improving the production of astaxanthin.

**Table 2:** Effect of CRISPR interference of the mutated genes on production of astaxanthin in *E. coli* AST-4

Gene	Protein	Amino acid mutation	Ratio of astaxanthin concentration^b^
Response to stress			
* yadC* (b0135)	Fimbrial tip-adhesin YadC	Gly39Asp	1.40 ± 0.01
* lon* (b0439)	Lon protease	Lys554fs^a^	1.22 ± 0.01
* cusB* (b0574)	Copper/silver export system membrane fusion protein	Pro103Ser	1.11 ± 0.05
* acnA* (b1276)	Aconitate hydratase 1	Ser522Gly	1.09 ± 0.01
* rcsC* (b2218)	Sensory histidine kinase RcsC	Phe136Leu	1.45 ± 0.07
Membrane and transport protein			
* pqiA* (b0950)	Intermembrane transport protein PqiA	Met163Ile	1.17 ± 0.08
* fixA* (b0041)	Putative electron transfer flavoprotein FixA	Ile137Thr	1.09 ± 0.05
Cellular lipid metabolic process			
* fabI* (b1288)	Enoyl-[acyl-carrier-protein] reductase	Tyr146His	1.06 ± 0.02
Genetic information processing			
* infA* (b0884)	Translation initiation factor IF-1	Lost of the start codon Met	1.19 ± 0.04
* fabR* (b3741)	Transcriptional repressor of *fabA* and *fabB*	Gly61Val	1.05 ± 0.08
* ygfI* (b2921)	DNA-binding transcriptional regulator YgfI	Ile85Thr	1.63 ± 0.01
* mnmG* (b3741)	Carboxymethylaminomethyluridine-tRNA synthase subunit MnmG	Gly311Asp	1.52 ± 0.03
* rppH* (b2830)	RNA pyrophosphohydrolase	Ser32Pro	1.21 ± 0.06
* rpoC* (b3988)	RNA polymerase subunit beta'	Ala1323Thr	1.17 ± 0.01
* sseA* (b2521)	3-mercaptopyruvate sulfurtransferase	Gly122fs	1.14 ± 0.03
* glpR (b3423)*	*Glycerol-3-phosphate regulon repressor*	*Ala51fs*	*1.12* ± *0.01*
Signaling and cellular processes			
* sfmF* (b0534)	Uncharacterized fimbrial-like protein SfmF	Glu127fs	1.21 ± 0.03
* phoH* (b1020)	ATP-binding protein PhoH	Cys314Arg	1.12 ± 0.12
Xenobiotics biodegradation and metabolism			
* hybO* (b2997)	Hydrogenase 2 small subunit	Pro291Ser	1.34 ± 0.01
Carbohydrate metabolism			
* frlB* (b3371)	Fructoselysine 6-phosphate deglycase	Ser140Gly	1.31 ± 0.01
* ydiF* (b1694)	Putative acetate-CoA transferase	Val22Met	1.30 ± 0.07
* ttdA* (b3061)	l (+)-tartrate dehydratase subunit alpha	Ile243Val	1.29 ± 0.01
Nucleotide metabolism			
* cpdA* (b3032)	cAMP phosphodiesterase	Tyr275Cys	1.11 ± 0.02
NO KO assigned			
* yadE* (b0130)	Uncharacterized protein, hydrolase activity	Asp380fs	1.22 ± 0.01
* icdC* (b4519)	Protein IcdC	Ser44fs, Glu45fs	1.18 ± 0.05
* ymcF* (b4723)	Protein YmcF	Thr19Ala	1.10 ± 0.09
* intQ* (b1579)	Qin prophage; putative defective integrase	Phe274Leu	1.09 ± 0.01
* yfcV* (b2339)	Putative fimbrial protein YfcV	Leu164Pro	1.46 ± 0.05
* pdeN* (b2176)	Phosphodiesterase PdeN	Val50Ala, Val141Met, Ala414Val	1.41 ± 0.01

^a^Frameshift variant

^b^The ratio of astaxanthin production with CRISPRi and without

**Fig. 6:**
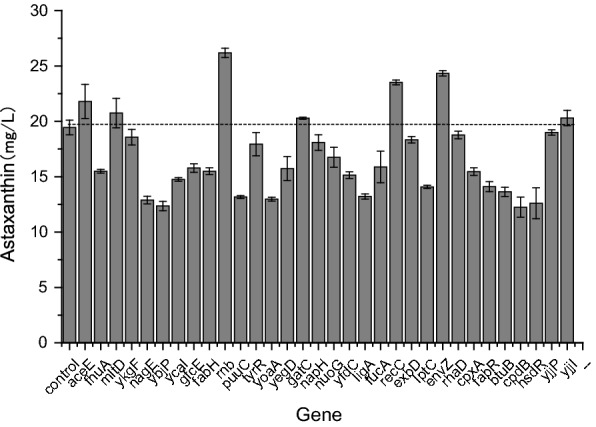
Effect of CRISPRa of genes on the production of astaxanthin in *E. coli* AST-4. *E. coli* AST-4 harboring pBbB2K-dCas9*-MCPSoxS and pTargetA-X was set as the control. The data represent the means of three replicates, and error bars indicate standard deviations

Other genes without positive effect after CRISPRi were selected to activate using CRISPR. The results are presented in Fig. 6. As shown in Fig. 6, the activations of *rnb, envZ, recC, aceE* and *mltD* increased the production of astaxanthin by 6–35%. *rnb* encodes exoribonuclease 2 involved in mRNA degradation, which hydrolyzes single-stranded polyribonucleotides processively in the 3′ to 5′ direction. *envZ* encodes sensor histidine kinase EnvZ, which is a member of the two-component regulatory system EnvZ/OmpR involved in osmoregulation and acid stress response. *recC* encodes exodeoxyribonuclease V gamma subunit, which is a helicase/nuclease that prepares dsDNA breaks (DSB) for recombinational DNA repair. *mltD* encodes membrane-bound lytic murein transglycosylase D, which is a murein-degrading enzyme. The activations of *envZ* and *mltD* may increase the stress tolerance of strain.

**Fig. 7:**
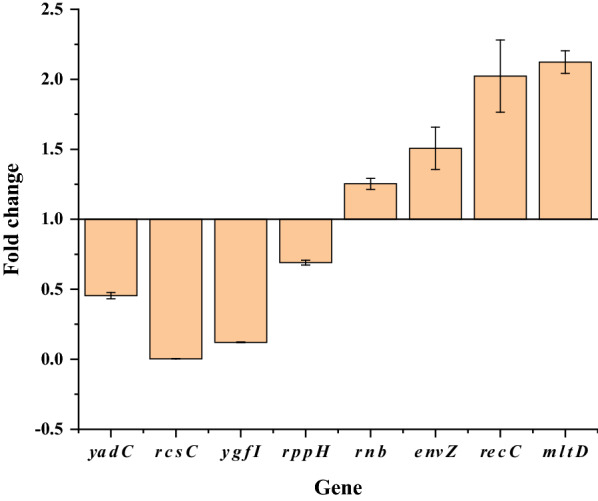
Relative transcriptional levels of the selected mutant genes in *E. coli* AST-4AS compared with those in *E. coli* AST-4

To investigate whether gene mutations caused changes in transcriptional levels in *E. coli* AST-4AS, the transcriptional levels of some activated and suppressed genes were determined using quantitative real-time PCR (qRT-PCR) and were compared with those in *E. coli* AST-4 (Fig. 7). The transcriptional levels of *rnb, envZ, recC* and *mltD* in *E. coli* AST-4AS were upregulated by 25%, 51%, 100% and 112%, respectively. The transcriptional levels of *rppH, yadC,ygfI* and *rcsC* in *E. coli* AST-4AS were downregulated by 31%, 55%, 88% and 99%, respectively. These results indicate that these gene mutations caused their changes in transcriptional levels, thereby improving the production of astaxanthin.

### Omics-guide genetic modification

**Fig. 8:**
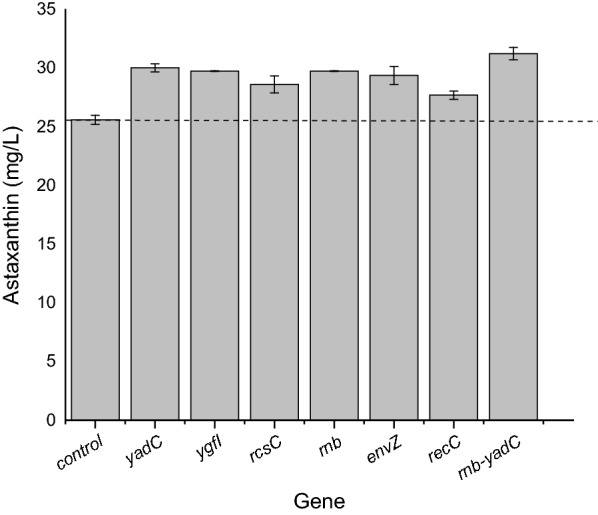
Effects of CRISPRi and CRISPRa of the selected genes on production of astaxanthin in *E. coli* AST-4AS. *E. coli* AST-4AS harboring pBbB2K-dCas9*-MCPSoxS and pTargetA-X was set as the control. CRISPRi for *yadC, ygfI* and *rcsC*; CRISPRa for *rnb, envZ* and *recC*. The data represent the means of three replicates, and error bars indicate standard deviations

To investigate whether the above identified genes have effects on the production of astaxanthin in in the genome shuffled strain *E. coli* AST-4AS, the mutated genes with the highest astaxanthin production after CRISPRi or CRISPRa, were tested in *E. coli* AST-4AS. As shown in Fig. 8, CRISPRi or CRISPRa of these genes further improved the production of astaxanthin in the genome shuffled strain *E. coli* AST-4AS. Repression of *yadC, ygfI* and *rcsC* increased the astaxanthin production by 17%, 16% and 12%, respectively. Activation of *rnb, envZ* and *recC* increased the astaxanthin production by 16%, 15% and 8%, respectively.

We also investigate the synergy effects of activated and repressed genes with the highest astaxanthin production in the genome shuffled strain *E. coli* AST-4AS. As shown in Fig. 8, the activation of *rnb* and the repression of *yadC* had a synergy effect on the production of astaxanthin in the genome shuffled strain *E. coli* AST-4AS. The combination of *rnb-yadC* increased the production of astaxanthin by 22%. *E. coli* AST-4 and AST-4AS harboring CRISPRi/a plasmids produced much lower astaxanthin than those without plasmids (Figs. 1, 2, 3, 6 and 8). The decreased astaxanthin production may result from the metabolic burden caused by plasmids. Moreover, a plasmid-based expression system also has other drawbacks, such as genetic instability because of plasmid instability and the requirement of the addition of antibiotics for constant selective pressure. Thus, the replacing of *yadC* with the *rnb* under the control of P37 promoter was performed in *E. coli* AST-4AS. The resulting strain *E. coli* AST-4ASΔyadC::P37-rnb produced astaxanthin of 78.63 ± 0.36 mg/L (Table 3), which is higher than that obtained by *E. coli* AST-4AS. This result indicates that simultaneous deletion of *yadC* and overexpression of *rnb* indeed further improved the production of astaxanthin. The effects of other targeted genes should be needed further to investigate.

**Table 3:** Effect of gene replacing on astaxanthin production

Strain	Astaxanthin (mg/L)
*E. coli* AST-4AS	59.57 ± 0.52
*E. coli* AST-4ASΔyadC::P37-rnb	78.63 ± 0.36

The above genes are difficult to be found by general rational analysis. These results demonstrated that the genes identified in this study can be used for further cell factory optimization.

## Conclusions

We developed an ALE strategy to enhance the production of chemicals by improving strain tolerance against industrial fermentation conditions. We applied this ALE strategy to improve *E. coli* tolerance, thereby enhancing the production of astaxanthin. A shuffled strain *E. coli* AST-4AS tolerant to the high cell density culture conditions (5 g/L NaAc, 0.3 M NaCl and 2 mM H_2_O_2_, pH 5.5) was obtained. The shuffled strain *E. coli* AST-4AS produced a 53.7% higher level of astaxanthin with 18.5% higher cell density in the fed-batch fermentation compared to the parent strain *E. coli* AST-4. Whole-genome resequencing shows that 65 mutations with amino acid substitution were identified in 61 genes of the shuffled strain *E. coli* AST-4AS. CRISPRi and CRISPRa reveal that the shuffled strain with higher astaxanthin production may be associated with the mutations of some stress response protein genes (*yadC, lon, cusB, acnA, rcsC*, *ygfI* and *envZ*), some fatty acid biosynthetic genes and *rppH*. Repression of *yadC, ygfI* and *rcsC*, activation of *rnb, envZ* and *recC* further improved the production of astaxanthin in the shuffled strain *E. coli* AST-4AS. Replacement of *yadC* with the *rnb* under the control of P37 promoter increased the production of astaxanthin by 32% in the shuffled strain *E. coli* AST-4AS. Our works also provide some valuable target genes, which are hardly found through rational analysis, to build cell factories for efficient astaxanthin production in the future. This ALE strategy based on tolerance engineering is powerful for constructing cell factories for the production of chemicals.

## Methods

### Strains and plasmids

The bacterial strains and plasmids used in this study are listed in Table 4. Primers used in this study are presented in Additional file 1: Table S2.

**Table 4:** Strains and plasmids used in this study

Strain	Description	Source /reference
*E. coli* AST-4	Astaxanthin producing strain, *E. coli* AST-2 Δ*lpp*Δ*bamB*Δ*uspE*Δ*yggE*	[17]
*E. coli* AST-4A	Astaxanthin producing strain, *E. coli* AST-4 mutant after the adaptive laboratory evolution	This study
*E. coli* AST-4AS	Astaxanthin producing strain, the shuffled strain of *E. coli* AST-4A after ep-WGS	This study
*E. coli* AST-4ASΔyadC::P37-rnb	Astaxanthin producing strain, replacement of *yadC* with the *rnb* under the control of P37 promoter in *E. coli* AST-4AS	This study
pSIM6	pSC101 replicon, P_L_-*gam-bet-exo*, Amp^r^	[40]
pBbB2K-dCas9*-MCPSoxS	CRISPRi/CRIPRa plasmid; pBbB2K-dCas9* containing the MCPSoxS sequences	[38]
pTargetA	*E. coli* scRNA expression vector, BglBrick vector, P_tet_ promoter, Spe^r^, pMB1 *ori*	[38]
pZSKBP	constitute expression vector, pSC101 ori, P37 promoter, kan^r^, BglBrick, ePathBrick containing four isocaudamer (*Avr*II, *Nhe*I, *Spe*I, and *Xba*I)	[17]

### ALE

*Escherichia coli* AST-4 was treated by an ARTP mutation system (ARTP-IIS, Tmaxtree Biotechnology Co, Ltd, Wuxi, China) as described by Niu et al. [34]. The parameters used were as follows: (1) the radio frequency power input was 100 W; (2) the flow of pure helium was 10 L/min; (3) the distance between the plasma torch nozzle exit and the slide was 2 mm; and (4) different treatment times were selected (20, 40, 50, 60, 70, 80, 100 and 120 s).

After ARTP treatment, adaptive evolution was performed as described by Niu et al. [34]. Briefly, the slide was washed with LB medium, transferred to 5 mL of LB medium in a 15 mL falcon tube, and cultivated at 37 °C and 200 rpm for 12 h. The cultures were serially passed into fresh LB medium supplemented with 1 g/L of NaAc, 0.06 M NaCl and 0.4 mM H_2_O_2_ (pH6.7) (initial OD_600_ of 0.05) every 12 h. After continuously repeating this transfer procedure until the OD_600_ at 12 h reached 3.0, the culture was then sequentially transferred to a fresh LB medium supplemented with a higher concentration of NaAc, NaCl and H_2_O_2_, and lower pH value. The ALE was carried out until the concentration of NaAc, NaCl and H_2_O_2_, and pH value reached 5 g/L, 0.3 M, 2 mM and 5.5, respectively. The cultures were frozen and stored at − 80 °C at every tolerance condition.

### ep-WGS

ep-WGS was carried out according to the protocols reported by Ye et al. [36] with some modifications. Genomic DNA of *E. coli* AST-4A was used for the epPCR templates. The error-prone whole genome amplification was carried out in a total volume of 50 µL, containing 100 ng genomic DNA, 33.3 μM 15-mer random primers, 0.2 mM each of dATP and dGTP, 1 mM each of dCTP and dTTP, 5 mM MgCl_2_, 0.3 mM MnCl_2_, and 2 U Taq DNA polymerase. The PCR reaction conditions were as follows: 92 °C for 1 min, 50 cycles at 37 °C for 2 min, a programmed ramping of 0.1 °C per sec to 55 °C, and 4 min extension at 55 °C. The amplified PCR products were ethanol-precipitated and stored at − 80 °C.

*Escherichia coli* AST-4A (pSIM46) were incubated in LB media with 100 μg/mL ampicillin at 30 °C and 200 rpm to the OD_600_ value of ca. 0.3–0.35. Then, the cultures were heat-shocked in a shaking water bath at 42 °C for 15 min to induce expression of the Red recombinase. The electro-competent cells were prepared. 100 μL competent cells were mixed with 200 ng of the above epPCR products and incubated in an ice bath for 5 min. Then, the electroporation was carried out at 2500 V in 1-mm gap cuvettes on the Electroporator MicroPulser™ 6600 (Bio-Rad). The suspensions were immediately mixed with 600 μL SOC (2% tryptone, 0.5% yeast extract, 0.05% NaCl, 0.0186% KCl, 0.095% MgCl_2_, 0.036% glucose) and transferred to a microcentrifuge tube for culture at 37 °C with 200 rpm for 3 h. All the culture medium was plated on LB agar with 100 μg/mL ampicillin and 1% glucose at 37 °C overnight and scraped off to create a liquid library.

### Library screening

Because astaxanthin but not the other carotenoids can efficiently diffuse through the cell membranes [41], a colorimetry of culture media (measured at 515 nm) was developed to be used for the screening of high astaxanthin-producing strains [15]. Cells from the library were diluted and then spread on LB plates. Single colonies with red color were inoculated in individual wells of a 48 deep-well microplate (4.6 mL) containing 1 mL of LB medium and incubated at 30 °C and 1000 rpm for 48 h on an MBR-420FL shaker (TAITEC, Japan). Two hundred microliter of the bacterial culture were centrifugated at 5000×*g* for 10 min. Then, the supernatant was transferred into a 96-well plate in which the OD_515_ was read using a SynergyNeo2 multi-mode reader (SynergyNeo2, BioTek, USA).

### Whole-genome resequencing and data analysis

Total genomic DNA of *E. coli E. coli* AST-4AS was extracted from mid-log phase bacterial cultures according to the manufacturer’s protocol using the TIANamp Bacterial DNA Kit (Tiangen Biotech Co., Beijing, China). Genomic library construction and whole-genome resequencing were performed on the Illumina NOVAseq platform by Sangon Biotech (Shanghai, China). The paired-end reads from *E. coli E. coli* AST-4AS were aligned to the reference genome of *E. coli* MG1655 using BWA software (Burrows-Wheeler Aligner, version 0.7.17). Mutations, including base substitutions, deletions, and insertions, were detected by SAMtools (version 1.9), MarkDuplicates (version 4.1.1.0), and BEDTools (version 2.28.0). DNA frameshift mutations were further validated by PCR and sequencing.

### CRISPRa and CRISPRi of the mutant genes

Mutant genes with CRISPR activation and interference were performed as described by Niu et al. [38]. The pTargetA-X series used in targeted single-gene activation or repression, with N20 sequences targeting gene loci of interest, was obtained by inverse PCR from pTargetA using primers targeting N20F/N20 genes, then cut with *Kpn*I and self-ligated. For activation, the N20 sequence was designed to target the non-template strand upstream of the promoter. For repression, the N20 sequence was designed to target the 5′ end (about 100 bp downstream of ATG) of the gene on the non-template DNA strand. As the scRNA fragment was flanked by *Bam*HI and *Bgl*II, the scRNA expressing vector pTargetA-XY could be reassembled from pTargetA-X and pTargetA-Y using the standard BglBrick assembly approach.

To investigate the effects of activation and repression on growth and astaxanthin production, pBbB2K-dCas9*-MCPSoxS and pTargetA-X were co-transferred into *E. coli* AST-4. A single colony was grown in 5 mL LB (with 1% glucose) in a falcon tube at 37 °C overnights. The overnight cultures were inoculated into 10 mL of the fermentation medium at 37 °C until the OD_600_ reached 0.8, anhydrous tetracycline was added to the media at a final concentration of 200 nM to induce the expression of dcas9*, and then further cultured for 72 h.

### Astaxanthin production

A single colony was inoculated into 5 mL of LB medium in a falcon tube, which was incubated overnight at 30 °C. The overnight seed culture was then inoculated into 50 mL of SBMSN medium with an initial OD_600_ of 0.1. SBMSN medium (pH 7.0) contained (per liter) 12 g peptone, 24 g yeast extract, 1.7 g KH_2_PO_4_, 11.42 g K_2_HPO_4_, 1 g MgCl_2_⋅6H_2_O, 1.42 g ammonium oxalate, and 2 g Tween-80. The cultures were incubated at 30 °C for 48 h in a rotary shaking incubator set to 150 rpm. Cell growth was measured according to the OD_600_ and converted into DCW (g/L) using a standard curve.

Fed-batch fermentation was performed in a 2 L fermenter (MiniBox 2 L*2 Parallel Bioreactor System, T&J Bioengineering (Shanghai) Co. LTD, Shanghai, China) containing 1.2 L of SBMSN medium with an initial OD_600_ of approximately 0.1. The temperature was controlled at 30 °C, and the pH value was maintained at 7.0 by the automatic addition of NH_4_OH. The airflow rate was 2 L/min. Dissolved oxygen was kept above 25% by adjusting the agitation rate from 400 to 1200 rpm. A feed solution (pH 7.0) containing (per liter) 500 g glucose, 15 g peptone, 30 g yeast extract and 30 g MgSO_4_·7H_2_O was fed continuously to the fermenter using a pH–stat feeding strategy. Once the glucose was depleted, the pH rose rapidly. When the pH was greater than 7.1, the feed was automatically added to the fermenter. Samples were periodically withdrawn, and these parameters (OD_600_ and astaxanthin concentrations) were determined.

### Quantitative real-time PCR (qRT-PCR)

The total RNA from *E. coli* cells grown in the fermentation medium for 36 h in shake flasks was isolated using an RNA extraction kit (Dongsheng Biotech, Guangzhou, China) following the manufacturer’s instructions. The first-strand cDNA was synthesized using an All-in-One™ First-Strand cDNA Synthesis kit (GeneCopoeia, Guangzhou, China). Quantitative real-time PCR was performed with the All-in-One™ qPCR Mix kit (GeneCopoeia) by an iCycler iQ5 Real Time PCR system (Bio-Rad Laboratories, California, USA). One hundred ng of cDNA was added as a template. The PCR program was set as follows: 95 °C for 10 min, followed by 45 cycles of denaturation at 95 °C for 10 s, annealing at 60 °C for 20 s, and extension at 72 °C for 15 s. The expression levels were analyzed by the 2^−ΔΔCt^ method described by Livak et al. [42] and normalized by *cysG* gene expression as reference. Three biological replicates for each sample were used for qRT-PCR analysis, and three technical replicates were analyzed for each biological replicate.

### Replacement of gene

Gene knockout or replacement was performed according to the CRISPR-Cas method as previously described [17, 34, 43, 44]. The sgRNA plasmid pTargetB-yadC for replacement was obtained as described above for the CRISPRi system. The *rnb* was amplified from *E. coli* by PCR and then inserted into *Kpn*I/*Apa*I-digested pZSKBP to obtain pZSKBP-rnb. The upstream and downstream homology arms of the *yadC* were amplified and then successively inserted into the *Mlu*I/*Avr*II and *Apa*I/*Sma*I of pZSKBP-rnb. The targeting fragment was cut from the above plasmid using *Mlu*I/*Sma*I and then transferred into the electrocompetent cells harboring pCas* and pTargetB-yadC to replace the corresponding gene.

### Extraction and quantification of carotenoids

Cells were extracted with acetone to isolate carotenoids as previously described [9, 17]. *E. coli* cultures (250 μL) were harvested by centrifugation at 13,523×*g* for 5 min. The cell pellet was washed with water and extracted with 1 mL of acetone at 55 °C for 15 min with intermittent vortexing. The acetone supernatant after centrifugation was transferred to a new tube. Carotenoids were analyzed by HPLC (Shimadzu HPLC system, Model LC-20A, Shimadzu, Japan) using an Inertsil ODS-SP column (5 μm, 4.6 × 150 mm, GL Sciences Inc., Tokyo, Japan). The mobile phase was acetonitrile–methanol (65:35 v/v) at a flow rate of 1 mL/min. The absorbance of carotenoids at 477 nm was detected using a photodiode array detector (SPD-M20A). Carotenoid compounds were identified based on their retention times relative to standard compounds (Sigma-Aldrich, St. Louis, MO, USA). Astaxanthin was quantified by comparing the integrated peak areas with those of authentic standards.

### Statistical analysis

All experiments were conducted in triplicate, and data were averaged and presented as the means ± standard deviation. Oneway analysis of variance followed by Tukey’s test was used to determine significant differences using the OriginPro (version 9.0) package. Statistical significance was defined as *p* < 0.05.

## Supplementary Information

**Additional file 1: Fig. S1.** The colorimetry of culture media for high throughput screening. **Fig. S2.** Production of astaxanthin determined using OD515 by the evolved strains after ARTP mutation. **Fig. S3.** Production of astaxanthin determined using OD515 by the strains after error-prone whole-genome shuffling. **Fig. S4.** HPLC analysis of carotenoid products extracted from *E. coli* AST-4AS cultured in 2-L bioreactor. **Fig. S5.** Effect of CRISPR repressing of the mutated gene on the astaxanthin production. **Table S1.** Mutated genes identified in *E. coli* AST-4AS. **Table S2.** Primers used in this study.

## Data Availability

Not applicable.
