# Supplementary material for: Adaptive laboratory evolution and shuffling of Escherichia coli to enhance its tolerance and production of astaxanthin
Source: Biotechnol Biofuels Bioprod. 2022 Feb 16;15:17. doi: 10.1186/s13068-022-02118-w (PMC8851715; doi:10.1186/s13068-022-02118-w)
Supplement: Supplementary file 1 — Additional file 1: Fig. S1. The colorimetry of culture media for high throughput screening. Fig. S2. Production of astaxanthin determined using OD515 by the evolved strains after ARTP mutation. Fig. S3. Production of astaxanthin determined using OD515 by the strains after error-prone whole-genome shuffling. Fig. S4. HPLC analysis of carotenoid products extracted from E. coli AST-4AS cultured in 2-L bioreactor. Fig. S5. Effect of CRISPR repressing of the mutated gene on the astaxanthin production. Table S1. Mutated genes identified in E. coli AST-4AS. Table S2. Primers used in this study. [file 13068_2022_2118_MOESM1_ESM.docx]

**Adaptive laboratory evolution and shuffling of *Escherichia coli* to enhance its tolerance and production of astaxanthin**

Qian Lu, Xiao-Ling Zhou and Jian-Zhong Liu^*^

Institute of Synthetic Biology, School of Life Sciences, Sun Yat-Sen University, Guangzhou 510275, China

* Corresponding author: Institute of Synthetic Biology, School of Life Science, Sun Yat-Sen University, Guangzhou 510275, P.R. China. Phone: +86-20-84110115. Fax: +86-20-84036461. *E-mail address*: lssljz@mail.sysu.edu.cn (J. Z. Liu)


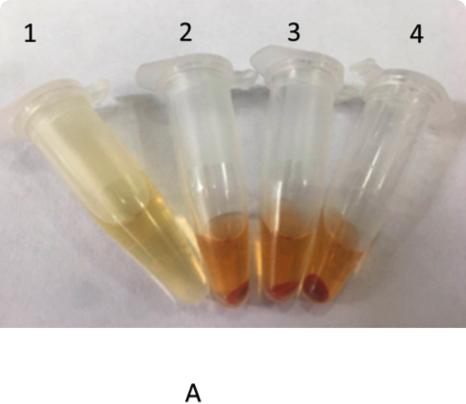


Fig. S1 The colorimetry of culture media for high throughput screening. (A) Photograph of the fermentation broth after centrifugation. 1: without astaxanthin producing strain (ie LB medium, OD_515_ was 0.018); 2-4: with *E. coli* strain with different astaxanthin titer. (B) The relationship between intracellular astaxanthin content and OD_515_ value

Fig. S2 Production of astaxanthin determined using OD515 by the evolved strains after ARTP mutation. Dashed line represents the OD515 value of the starting strain *E. coli* AST-4. The OD_515_ value of all strains was significantly higher than that (0.018) of the LB medium only (*p<*0.01).

Fig. S3 Production of astaxanthin determined using OD515 by the strains after error-prone whole-genome shuffling. Dashed line represents the OD515 value of the starting strain *E. coli* AST-4A. The OD_515_ value of all strains was significantly higher than that (0.018) of the LB medium only (*p<*0.01).

Fig. S4 HPLC analysis of carotenoid products extracted from *E. coli* AST-4AS cultured in 2-L bioreactor

A

B

Fig. S5 Effect of CRISPR repressing of the mutated gene on the astaxanthin production. *E. coli* AST-4 containing pBbB2K-dCas9*-MCPSoxS and pTargetA was set as the control.

Table S1 Mutated genes identified in *E. coli* AST-4AS

| GENE | | Des. | Mutation type | Amino acid mutation | KO. |
| --- | --- | --- | --- | --- | --- |
| **Cellular Processes** | | | | | |
| **Cell growth and death** | | |  |  |  |
| *lon* | b0439 | Lon protease | frameshift_variant | p.Lys554fs | K01338-ko04112 Cell cycle - Caulobacter |
| **Environmental Information Processing** | | | | | |
| **Membrane transport** | | |  |  |  |
| *nagE* | b0679 | N-acetylglucosamine specific PTS enzyme IIABC component | missense_variant | p.Leu357Ser | ko02060 Phosphotransferase system (PTS) |
| **Signal transduction** | | |  |  |  |
| *cusB* | b0574 | copper/silver export system membrane fusion protein | missense_variant | p.Pro103Ser | K07798-ko02020 Two-component system |
| *rcsC* | b2218 | sensory histidine kinase RcsC | missense_variant | p.Phe136Leu | K07798-ko02020 Two-component system |
| *envZ* | b3404 | sensory histidine kinase EnvZ | frameshift_variant | p.Tyr89fs | K07798-ko02020 Two-component system |
| *cpxA* | b3911 | sensory histidine kinase CpxA | missense_variant | p.Thr89Ala | K07798-ko02020 Two-component sys |
| **Folding sorting and degradation** | | |  |  |  |
| *rppH* | b2830 | RNA pyrophosphohydrolase | missense_variant | p.Ser32Pro | K08311-ko03018 RNA degradation |
| *sseA* | b2521 | 3-mercaptopyruvate sulfurtransferase | frameshift_variant | p.Gly122fs | K01011-ko04122 Sulfur relay system |
| **Replication and repair** | | |  |  |  |
| *ligA* | b2411 | DNA ligase | missense_variant | p.Ala479Thr | K01972-ko03420 Nucleotide excision repair |
| *ligA* | b2411 | DNA ligase | missense_variant | p.Ala479Thr | ko03410 Base excision repair |
| *ligA* | b2411 | DNA ligase | missense_variant | p.Ala479Thr | ko03030 DNA replication |
| *ligA* | b2411 | DNA ligase | missense_variant | p.Ala479Thr | ko03430 Mismatch repair |
| *recC* | b2822 | exodeoxyribonuclease V subunit RecC | missense_variant | p.Val986Ala | K03583-ko03440 Homologous recombination |
| **Genetic Information Processing** | | | | | |
| **Transcription** | | |  |  |  |
| *rpoC* | b3988 | RNA polymerase subunit beta' | missense_variant | p.Ala1323Thr | ko03020 RNA polymerase |
| **Metabolism** | | | | | |
| **Amino acid metabolism** | | |  |  |  |
| *puuC* | b1300 | gamma-glutamyl-gamma-aminobutyraldehyde dehydrogenase | missense_variant | p.Phe318Cys | K09472-ko00330 Arginine and proline metabolism |
| *sseA* | b2521 | 3-mercaptopyruvate sulfurtransferase | frameshift_variant | p.Gly122fs | K01011-ko00270 Cysteine and methionine metabolism |
| **Carbohydrate metabolism** | | |  |  |  |
| *acnA* | b1276 | aconitate hydratase 1 | missense_variant | p.Ser522Gly | K01681-ko00020 Citrate cycle (TCA cycle);ko01230 Biosynthesis of amino acids;ko01200 Carbon metabolism;ko01210 2-Oxocarboxylic acid metabolism;ko00630 Glyoxylate and dicarboxylate metabolism;ko00720 Carbon fixation pathways in prokaryotes |
| *aceE* | b0114 | pyruvate dehydrogenase E1 component | missense_variant | p.Arg607Leu | K00163-ko00010 Glycolysis / Gluconeogenesis;ko00020 Citrate cycle (TCA cycle);ko01200 Carbon metabolism;ko00620 Pyruvate metabolism |
| *ttdA* | b3061 | L(+)-tartrate dehydratase subunit alpha | missense_variant | p.Ile243Val | K03779-ko00630 Glyoxylate and dicarboxylate metabolism |
|  |  |  |  |  |  |
| *fucA* | b2800 | L-fuculose-phosphate aldolase | missense_variant | p.Glu215Gly | K01628-ko00051 Fructose and mannose metabolism |
| *rhaD* | b3902 | rhamnulose-1-phosphate aldolase | missense_variant | p.Gly168Asp | K01629-ko00040 Pentose and glucuronate interconversions;ko00051 Fructose and mannose metabolism |
| *ydiF* | b1694 | putative acetate-CoA transferase | missense_variant | p.Val22Met | K19709-ko00650 Butanoate metabolism;ko00640 Propanoate metabolism |
| *nagE* | b0679 | N-acetylglucosamine specific PTS enzyme IIABC component | missense_variant | p.Leu357Ser | K02804-ko00520 Amino sugar and nucleotide sugar metabolism |
| **Energy metabolism** | | |  |  |  |
| *sseA* | b2521 | 3-mercaptopyruvate sulfurtransferase | frameshift_variant | p.Gly122fs | K01011-ko00920 Sulfur metabolism |
| *acnA* | b1276 | aconitate hydratase 1 | missense_variant | p.Ser522Gly | K01681ko00720 Carbon fixation pathways in prokaryotes |
| *nuoG* | b2283 | NADH:quinone oxidoreductase subunit G | missense_variant | p.Ala627Gly | K00336-ko00190 Oxidative phosphorylation |
| **Lipid metabolism** | | |  |  |  |
| *fabH* | b1091 | beta-ketoacyl-[acyl carrier protein] synthase III | missense_variant | p.Asn210Ser | ko00061 Fatty acid biosynthesis |
| *fabI* | b1288 | enoyl-[acyl-carrier-protein] reductase | missense_variant | p.Tyr146His | ko00061 Fatty acid biosynthesis |
| **Metabolism of cofactors and vitamins** | | | |  |  |
| *fabI* | b1288 | enoyl-[acyl-carrier-protein] reductase | missense_variant | p.Tyr146His | ko00780 Biotin metabolism |
| **Nucleotide metabolism** | | |  |  |  |
| *cpdA* | b3032 | cAMP phosphodiesterase | missense_variant | p.Tyr275Cys | K03651 |
| *cpdB* | b4213 | 2'3' cyclic nucleotide phosphodiesterase/3' nucleotidase | missense_variant | p.Ala599Val | K01119-ko00230 Purine metabolism;ko00240 Pyrimidine metabolism |
| *rpoC* | b3988 | RNA polymerase subunit beta' | missense_variant | p.Ala1323Thr | K03046-ko00230 Purine metabolism;ko00240 Pyrimidine metabolism |
| **Overview** | | |  |  |  |
| *acnA* | b1276 | aconitate hydratase 1 | missense_variant | p.Ser522Gly | ko01230 Biosynthesis of amino acids;ko01200 Carbon metabolism; |
| *aceE* | b0114 | pyruvate dehydrogenase E1 component | missense_variant | p.Arg607Leu | ko01230 Biosynthesis of amino acids |
| **Xenobiotics biodegradation and metabolism** | | | |  |  |
| *hybO* | b2997 | hydrogenase 2 small subunit | missense_variant | p.Pro291Ser | K06282-ko00633 Nitrotoluene degradation |
| *ydiF* | b1694 | putative acetate-CoA transferase | missense_variant | p.Val22Met | K19709-ko00627 Aminobenzoate degradation |
|  |  |  |  |  |  |
| **Brite Hierarchies** | | | | | |
| **Protein families: signaling and cellular processes** | | | |  |  |
| *fhuA* | b0150 | ferrichrome outer membrane transporter/phage receptor | stop_gained | p.Ser241* | K02014 |
| *sfmF* | b0534 | putative fimbrial protein SfmF | frameshift_variant | p.Glu127fs | K07355 |
| *ycaI* | b0913 | conserved inner membrane protein YcaI | missense_variant | p.Glu204Gly | K02238 |
| *gfcE* | b0983 | putative exopolysaccharide export lipoprotein GfcE | missense_variant | p.Asp41Gly | K01991 |
| *phoH* | b1020 | ATP-binding protein PhoH | missense_variant | p.Cys314Arg | K06217 |
| *exbD* | b3005 | Ton complex subunit ExbD | missense_variant | p.Gln57Leu | K03559 |
| *lptC* | b3199 | lipopolysaccharide transport system protein LptC | missense_variant | p.Asp89Gly | K11719 |
| *btuB* | b3966 | cobalamin/cobinamide outer membrane transporter | missense_variant | p.Ala162Gly | K16092 |
| *hsdR* | b4350 | type I restriction enzyme EcoKI endonuclease component | stop_gained | p.Gln428* | K01153 |
| **Protein families: metabolism** | | | |  |  |
| *mltD* | b0211 | membrane-bound lytic murein transglycosylase D | missense_variant | p.Pro130Ser | K08307 |
| **Protein families: genetic information processing** | | | |  |  |
| *lacI* | b0345 | DNA-binding transcriptional repressor LacI | missense_variant | p.Ala331Val | K02529 |
| *infA* | b0884 | translation initiation factor IF-1 | start_lost | p.Met1? | K02518 |
| *tyrR* | b1323 | DNA-binding transcriptional dual regulator TyrR | missense_variant | p.Val209Ile | K03721 |
| *yegD* | b2069 | HSP70 family protein YegD | missense_variant | p.Ser92Pro | K04046 |
| *gatC* | b2092 | galactitol-specific PTS enzyme IIC component | frameshift_variant | p.Gly306fs | K02435 |
| *glpR* | b3423 | DNA-binding transcriptional repressor GlpR | frameshift_variant | p.Ala51fs | K02444 |
| *mnmG* | b3741 | 5-carboxymethylaminomethyluridine-tRNA synthase subunit MnmG | missense_variant | p.Gly311Asp | K03495 |
| *fabR* | b3963 | DNA-binding transcriptional repressor FabR | missense_variant | p.Gly61Val | K22105 |
| **Not Included in Pathway or Brite** | | | | | |
| **Unclassified: metabolism** | | | |  |  |
| *fixA* | b0041 | putative electron transfer flavoprotein FixA | missense_variant | p.Ile137Thr | K03521 |
| *ykgF* | b0307 | putative amino acid dehydrogenase with NAD(P)-binding domain and ferridoxin-like domain | missense_variant | p.Arg375His | K18929 |
| *frlB* | b3371 | fructoselysine 6-phosphate deglycase | missense_variant | p.Ser140Gly | K10708 |
| **Poorly characterized** | | | |  |  |
| *pqiA* | b0950 | intermembrane transport protein PqiA | missense_variant | p.Met163Ile | K03808 |
| *napH* | b2204 | ferredoxin-type protein NapH | missense_variant | p.Val63Ala | K02574 |
| Unclassified: signaling and cellular processes | | | |  |  |
| *yfdC* | b2347 | inner membrane protein YfdC | missense_variant | p.Ala207Val | K21990 |
| **no KO assigned** | | | | | |
| *yadE* | b0130 | putative polysaccharide deacetylase lipoprotein | missense_variant | p.Gly241Ser | - |
| *yadE* | b0130 | putative polysaccharide deacetylase lipoprotein | frameshift_variant | p.Asp380fs | - |
| *yadC* | b0135 | fimbrial tip-adhesin YadC | missense_variant | p.Gly39Asp | - |
| *ybjP* | b0865 | DUF3828 domain-containing lipoprotein YbjP | missense_variant | p.Val22Ala | - |
| *ymcF* | b4723 | protein YmcF | missense_variant | p.Thr19Ala | - |
| *icdC* | b4519 | protein IcdC | frameshift_variant | p.Ser44fs | - |
| *icdC* | b4519 | protein IcdC | frameshift_variant | p.Glu45fs | - |
| *intQ* | b1579 | Qin prophage; putative defective integrase | missense_variant | p.Phe274Leu | - |
| *yoaA* | b1808 | putative 5' to 3' DNA helicase implicated in DNA repair | frameshift_variant | p.Gly120fs | - |
| *pdeN* | b2176 | putative c-di-GMP phosphodiesterase PdeN | missense_variant | p.Val50Ala | - |
| *pdeN* | b2176 | putative c-di-GMP phosphodiesterase PdeN | missense_variant | p.Val141Met | - |
| *pdeN* | b2176 | putative c-di-GMP phosphodiesterase PdeN | missense_variant | p.Ala414Val | - |
| *yfcV* | b2339 | putative fimbrial protein YfcV | missense_variant | p.Leu164Pro | - |
| *ygfI* | b2921 | putative LysR-type DNA-binding transcriptional regulator YgfI | missense_variant | p.Ile85Thr | - |
| *yjjP* | b4364 | putative succinate exporter YjjP | stop_gained | p.Trp117* | - |
| *yjjI* | b4380 | DUF3029 domain-containing protein YjjI | missense_variant | p.Leu133Ile | - |

Table S2 Primers used in this study

| **Target Gene** | **5’-3’ Sequence** | |
| --- | --- | --- |
| CRISPRi |  | |
| *fixA* | ATACTAGT *accgcaatatcctgttcatc*GTTTTAGAGCTAGAAATAGCAAG | |
| *aceE* | ATACTAGT *tcgatcggatccacgtcatt* GTTTTAGAGCTAGAAATAGCAAG | |
| *yadE* | ATACTAGT *taacgcggcactgacacttg* GTTTTAGAGCTAGAAATAGCAAG | |
| *yadC* | ATACTAGT *tcatctgtgcggcagtggag* GTTTTAGAGCTAGAAATAGCAAG | |
| *fhuA* | ATACTAGT *agacatgccgctaaccgctg* GTTTTAGAGCTAGAAATAGCAAG | |
| *mltD* | ATACTAGT gcaacccacgagcaggacag GTTTTAGAGCTAGAAATAGCAAG | |
| *ykgF* | ATACTAGT tcggatcttcaatttgctga GTTTTAGAGCTAGAAATAGCAAG | |
| *lon* | ATACTAGT catcgcgcagcggcaatacg GTTTTAGAGCTAGAAATAGCAAG | |
| *sfmF* | ATACTAGT agggtcaactgcccatccac GTTTTAGAGCTAGAAATAGCAAG | |
| *cusB* | ATACTAGT cggttccgcctttgcaaccc GTTTTAGAGCTAGAAATAGCAAG | |
| *nagE* | ATACTAGT gccaccggcagcaccgcgat GTTTTAGAGCTAGAAATAGCAAG | |
| *ybjP* | ATACTAGT cactgaggagcagtgcgcag GTTTTAGAGCTAGAAATAGCAAG | |
| *infA* | ATACTAGT tctacgcggaacatggtatt GTTTTAGAGCTAGAAATAGCAAG | |
| *ycaI* | ATACTAGT aattggggcaaaatcagcaa GTTTTAGAGCTAGAAATAGCAAG | |
| *pqiA* | ATACTAGT gcatgtcacactgcgagcac GTTTTAGAGCTAGAAATAGCAAG | |
| *gfcE* | ATACTAGT ataaattctgaccaggaaca GTTTTAGAGCTAGAAATAGCAAG | |
| *ymcF* | ATACTAGT tcacatcgaatgccgatgtg GTTTTAGAGCTAGAAATAGCAAG | |
| *phoH* | ATACTAGT agtgacgagatgactaccgg GTTTTAGAGCTAGAAATAGCAAG | |
| *fabH* | ATACTAGT ggcgtttgtccgcacttgtt GTTTTAGAGCTAGAAATAGCAAG | |
| *icdC* | ATACTAGT acgttcgaagtcataagtta GTTTTAGAGCTAGAAATAGCAAG | |
| *acnA* | ATACTAGT cgcgcttttggacatggttg GTTTTAGAGCTAGAAATAGCAAG | |
| *rnb* | ATACTAGT cccagtgatttagcagcaag GTTTTAGAGCTAGAAATAGCAAG | |
| *fabI* | ATACTAGT cccttcagcgcgtggcgtct GTTTTAGAGCTAGAAATAGCAAG | |
| *puuC* | ATACTAGT catcgcctgagcgataccgt GTTTTAGAGCTAGAAATAGCAAG | |
| *tyrR* | ATACTAGT attcaccgttaataaataag GTTTTAGAGCTAGAAATAGCAAG | |
| *intQ* | ATACTAGT actccagttcagcaaaattg GTTTTAGAGCTAGAAATAGCAAG | |
| *ydiF* | ATACTAGT acagttacagccgatctccc GTTTTAGAGCTAGAAATAGCAAG | |
| *yoaA* | ATACTAGT gcttcctgtgccgacaggac GTTTTAGAGCTAGAAATAGCAAG | |
| *yegD* | ATACTAGT gctaccgccatctgtcgctg GTTTTAGAGCTAGAAATAGCAAG | |
| *gatC* | ATACTAGT tcgcttaccgcttcacgcgt GTTTTAGAGCTAGAAATAGCAAG | |
| *pdeN* | ATACTAGT gcagcatcaccgtagggccg GTTTTAGAGCTAGAAATAGCAAG | |
| *napH* | ATACTAGT ccagcacgaagaactgacaa GTTTTAGAGCTAGAAATAGCAAG | |
| *rcsC* | ATACTAGT ccagcacgaagaactgacaa GTTTTAGAGCTAGAAATAGCAAG | |
| *nuoG* | ATACTAGT agcaaaagtaaggaatatca GTTTTAGAGCTAGAAATAGCAAG | |
| *yfcV* | ATACTAGT agaacacactgagtcttcaa GTTTTAGAGCTAGAAATAGCAAG | |
| *yfdC* | ATACTAGT ccatcgcccgggaggggagt GTTTTAGAGCTAGAAATAGCAAG | |
| *ligA* | ATACTAGT tattcagcgtcgggaatttc GTTTTAGAGCTAGAAATAGCAAG | |
| *sseA* | ATACTAGT aatatgttcggcgagccagt GTTTTAGAGCTAGAAATAGCAAG | |
| *fucA* | ATACTAGT tgctttgataggctgccata GTTTTAGAGCTAGAAATAGCAAG | |
| *recC* | ATACTAGT atatcccagataaagctcgc GTTTTAGAGCTAGAAATAGCAAG | |
| *rppH* | ATACTAGT caaatcacgatacctacgtt GTTTTAGAGCTAGAAATAGCAAG | |
| *ygfI* | ATACTAGT tgccgcccgggcaatattat GTTTTAGAGCTAGAAATAGCAAG | |
| *hybO* | ATACTAGT aaagcttcatgaaatcacga GTTTTAGAGCTAGAAATAGCAAG | |
| *exbD* | ATACTAGT cgggcgcggctgcggcgtgc GTTTTAGAGCTAGAAATAGCAAG | |
| *cpdA* | ATACTAGT ttgtgcaaacaggtgagtgt GTTTTAGAGCTAGAAATAGCAAG | |
| *ttdA* | ATACTAGT caccacgtcatcaggcattc GTTTTAGAGCTAGAAATAGCAAG | |
| *lptC* | ATACTAGT cgatagcttagtgccccttc GTTTTAGAGCTAGAAATAGCAAG | |
| *frlB* | ATACTAGT cgcgttgagtggcgatccgc GTTTTAGAGCTAGAAATAGCAAG | |
| *envZ* | ATACTAGT gacgatgagcaataacgtac GTTTTAGAGCTAGAAATAGCAAG | |
| *glpR* | ATACTAGT gcgaatagtctgcgggctga GTTTTAGAGCTAGAAATAGCAAG | |
| *mnmG* | ATACTAGT ctgttgacccatacgcgccg GTTTTAGAGCTAGAAATAGCAAG | |
| *rhaD* | ATACTAGT tggtggctttgatcattccc GTTTTAGAGCTAGAAATAGCAAG | |
| *cpxA* | ATACTAGT cgccagcgtcagccagaaga GTTTTAGAGCTAGAAATAGCAAG | |
| *fabR* | ATACTAGT cggcttccaccagcgaacgg GTTTTAGAGCTAGAAATAGCAAG | |
| *btuB* | ATACTAGT tgctgcgcggctgttcaaaa GTTTTAGAGCTAGAAATAGCAAG | |
| *rpoC* | ATACTAGT cttcaccgaaagaccatgaa GTTTTAGAGCTAGAAATAGCAAG | |
| *cpdB* | ATACTAGT cattgcgggcatcgttaatc GTTTTAGAGCTAGAAATAGCAAG | |
| *hsdR* | ATACTAGT cagaccaagatgtttcgctg GTTTTAGAGCTAGAAATAGCAAG | |
| *yjjP* | ATACTAGT gcaaaagaaataatccacac GTTTTAGAGCTAGAAATAGCAAG | |
| *yjjI* | ATACTAGT ctgggctggtgacaatttgc GTTTTAGAGCTAGAAATAGCAAG | |
| CRISPRa |  | |
| target N20F | aagggtaccttctctatcactg | |
| *aceE* | CGGGGTACCagaaggcgacaaagcctctagttttagagctagaaatag | |
| *fhuA* | CGGGGTACCaacaaccagatgaaaagaaagttttagagctagaaatag | |
| *mltD* | CGGGGTACCaaccatgcaaaacgctcttcgttttagagctagaaatag | |
| *ykgF* | CGGGGTACCataactaattttatttctttgttttagagctagaaatag | |
| *nagE* | CGGGGTACCttattatcactcccttttacgttttagagctagaaatag | |
| *ybjP* | CGGGGTACC gatcgaaggcttagcaaacagttttagagctagaaatag | |
| *ycaI* | CGGGGTACC tctttaccaggtttaaagtggttttagagctagaaatag | |
| *gfcE* | CGGGGTACC gtaatgttatgaattacgtcgttttagagctagaaatag | |
| *fabH* | CGGGGTACC acagacaggcgccgttatacgttttagagctagaaatag | |
| *rnb* | CGGGGTACC tagcgcgctcaacagaatgagttttagagctagaaatag | |
| *puuC* | CGGGGTACC atatgcagtttgtgaacgtcgttttagagctagaaatag | |
| *tyrR* | CGGGGTACC aacaatcattgacacaaagagttttagagctagaaatag | |
| *yoaA* | CGGGGTACC gtgacggcgacctcacattggttttagagctagaaatag | |
| *yegD* | CGGGGTACC aatgttgccgtcgcgacaacgttttagagctagaaatag | |
| *gatC* | CGGGGTACC actacgatccactttggcaggttttagagctagaaatag | |
| *napH* | CGGGGTACC tttttcgcacttaccgcaacgttttagagctagaaatag | |
| *nuoG* | CGGGGTACC cagcgctctttcagcaggttgttttagagctagaaatag | |
| *yfdC* | CGGGGTACC atagaatcaagtagcctacagttttagagctagaaatag | |
| *ligA* | CGGGGTACC atttctgcggagtcatcatagttttagagctagaaatag | |
| *fucA* | CGGGGTACC cttaaaaatatttatcaaaagttttagagctagaaatag | |
| *recC* | CGGGGTACC ccgcctgcgatgtctgcattgttttagagctagaaatag | |
| *exbD* | CGGGGTACC ccgcaggaatcgctgccacggttttagagctagaaatag | |
| *lptC* | CGGGGTACC cagtggatgcgcatcggccagttttagagctagaaatag | |
| *envZ* | CGGGGTACC tcgcgggagagcggctcacggttttagagctagaaatag | |
| *rhaD* | CGGGGTACC gccgacctgtaggcctgatagttttagagctagaaatag | |
| *cpxA* | CGGGGTACC ttcctggcttaaatgttcacgttttagagctagaaatag | |
| *fabR* | CGGGGTACC aaaaatccaataaaacgtcagttttagagctagaaatag | |
| *btuB* | CGGGGTACC tccagacgtagctcacaaatgttttagagctagaaatag | |
| *cpdB* | CGGGGTACC aatgatgacactatcacagtgttttagagctagaaatag | |
| *hsdR* | CGGGGTACC tgaacctgttctgcgttatcgttttagagctagaaatag | |
| *yjjP* | CGGGGTACC atcataatgaatttattgttgttttagagctagaaatag | |
| *yjjI* | CGGGGTACC aattttattggcgacaagccgttttagagctagaaatag | |
| qPCR |  | |
| *yadC-F* | tataaacccacgcagctcgg | |
| *yadC-R* | aggattcgctctgccagatg | |
| *rcsC-F* | cgcttaccggaccagaaagt | |
| *rcs-R* | cgatgcttagcgatgagggt | |
| *ygfI-F* | taactcagggcgtcgttcac | |
| *ygfI-R* | ttcgctgaactcaactggct | |
| *rppH-F* | gcggcgatgcagaaatcaat | |
| *rppH-R* | acctgtctgaccggatacca | |
| *rnb-F* | tgaacccggtcctcactttg | |
| *rnb-R* | tcagcagacggtggttgatc | |
| *envZ-F* | actcggtgaggtgattgctg | |
| *envZ-R* | gcgttgaccaccatattcgc | |
| *recC-F* | accgcgccaatctctatcag | |
| *recC-R* | tgtttacccagcgcctgtag | |
| *mltD-F* | aacaaagcgcgtgggaaatc | |
| *mltD-R* | gtacgccataacgcttgctg | |
| *cysG-F* | atcaggccgcgactattcag | |
| *cysG-R* | ttcatttgctgcgccagttc | |
| Replacement of *yadC* with the *rnb* under the control of the P37 promoter | | |
| *yadC*-up-dF-*Mul*Ⅰ | | CGACGCGTccagggtaatattgatcttggcg |
| *yadC*-up-dR-*Avr*II | | CGCCTAGGttttactccaatgtttctttattgttatggtttg |
| *yadC*-dn-dF-*Apa*Ⅰ | | GAGGGCCC ttacgcgaccgggcgcgc |
| *yadC*-dn-dR-*Sma*Ⅰ | | TCCCCCGGG acgttcggtcagcatttgtac |
| *rnb*-UTR-F-*Kpn*Ⅰ | | GGGGTACCtttcggaattaaggaggtaataaatatgtttcaggacaacccgc |
| *rnb*-R-ApaI | | GAGGGCCC ttacgcgaccgggcgcgc |
| sgRNA for replacement of *yadC* | | |
| Target*yadC*-F | | GGACTAGTtcatctgtgcggcagtggagGTTTTAGAGCTAGAAATAG |
| TargetFR | | GGACTAGTATTATACCTAGGACTGAGCTAGCTGTCAAG |
